# Health Tract, No. 170

**Published:** 1863-09

**Authors:** 


HEALTH TRACT, No. 170.

### THE MONTH MALIGN.

SEPTEMBER gives rise to more disease in town and country together than any other month of the year. It is fruitful in diarrhea, dysentery, and fevers of every grade, from common fever and ague to the most malignant form of bilious, congestive, and yellow fever. The immediate causes of these maladies are the hot days and cool nights, in conjunction with the habits of the people. Few persons have hearty appetites in hot weather—our instincts are too wide awake for that; but we too often drown their wise, and steady, and gentle monitions in the clamor of the animal nature for stimulants, to whet up the appetite to hurtful and destructive activities. The proprietors of the most fashionable hotels in New-York have asserted that if it were not for the "profits of the bar" they would have to close their doors. Doubtless, in almost all cases, these "profits of the bar" are a very important source of income to all taverns. We have certainly noticed that a number of temperance hotels succeed in collapsing in a very short time. When the stomach is taxed beyond its ability of work, by eating to the fill of a stimulated appetite, one pernicious result always follows, and a different one is impossible in any single case in a century of centuries; that food is not perfectly assimilated; can not be made into good blood, and that, being mixed with what was already in the system, makes "bad blood" of the whole. The entire mass is a vitiated article, and becomes more so by each act of over-eating, by every mouthful swallowed to "get up an appetite." The whole mass of blood being thus corrupted, it is no wonder that persons living thus are liable to complaints in all parts of the body, for this vitiated blood goes everywhere; and never feeling well, they are always "taking something." In this way the body soon loses its vigor, its capability of resisting causes of disease, and warding off sickness; a state of things plainly proven and unwittingly acknowledged in the now very common expression: "The slightest thing in the world gives me a cold." When such is the case, it is always because the person so speaking has not much stamina; in other words, is full of "bad blood," whatever may have been the cause, whether from taking tonics, stimulants, or bitters, to wake up an unnatural appetite, or whether from "forcing" food; eating without an appetite; or merely from a vicious indulgence of the animal nature. When persons have for some time eaten more than the system requires, they lose their appetite; have a bad taste in the mouth on waking up in the morning; are more or less uncomfortably chilly, and are fit subjects for any cause of disease which may exist in the atmosphere; and they are the very first victims to any epidemic malady; if any body is sick, they are sure to be among the number. This general cause of disease existing in the atmosphere is always generated in the latter part of August and during September; it is called miasm—an emanation from decaying vegetable matter, mud, leaves, plants, roots, etc.; it is distilled death, literally, because the heat of the noonday sun acting upon matters like these, causes the deleterious agency to rise up, like alcohol or whisky from a still; when the cool of the evening comes, this air is condensed, becomes heavy, falls to the surface and is breathed by whole communities, sometimes breaking out in a night and destroying hundreds before the morning. In such cases the temperate, plain living, and industrious are the very last to suffer, if at all, because they have good blood, which has a "power" to resist disease. The lesson is, never attempt to "whet up" the appetite, except by creditable labor, or moderate, steady, continuous out-door activities.

